# Role of Circulating Biomarkers in Diabetic Cardiomyopathy

**DOI:** 10.3390/biomedicines12092153

**Published:** 2024-09-23

**Authors:** Raluca Diana Ianoș, Angela Cozma, Roxana Liana Lucaciu, Adriana Corina Hangan, Vasile Negrean, Delia Corina Mercea, George Ciulei, Călin Pop, Lucia Maria Procopciuc

**Affiliations:** 1 Department of Cardiology, “Iuliu Hațieganu” University of Medicine and Pharmacy, 400001 Cluj-Napoca, Romania; ralu_yannosh@yahoo.com; 2 4th Department of Internal Medicine, “Iuliu Hațieganu” University of Medicine and Pharmacy, 400015 Cluj-Napoca, Romania; vasile.negrean@umfcluj.ro (V.N.); george.ciulei@umfcluj.ro (G.C.); 3 Department of Pharmaceutical Biochemistry and Clinical Laboratory, Faculty of Pharmacy, “Iuliu-Hațieganu” University of Medicine and Pharmacy, 400012 Cluj-Napoca, Romania; liana.lucaciu@umfcluj.ro; 4 Department of Inorganic Chemistry, Faculty of Pharmacy, “Iuliu-Hațieganu” University of Medicine and Pharmacy, 400012 Cluj-Napoca, Romania; adriana.hangan@umfcluj.ro; 5 Department of Cardiology, Emergency County Hospital, 430031 Baia Mare, Romania; dancorinadelia@yahoo.com (D.C.M.); medicbm@yahoo.com (C.P.); 6 Faculty of Medicine Arad, “Vasile Goldis” Western University, 310045 Arad, Romania; 7 Department of Medical Biochemistry, University of Medicine and Pharmacy “Iuliu Hațieganu”, 400349 Cluj-Napoca, Romania; lprocopciuc@umfcluj.ro

**Keywords:** biomarkers, diabetic cardiomyopathy, heart failure, prognostic

## Abstract

Type 2 diabetes mellitus (T2DM) is a metabolic disorder that has alarmingly increased in incidence in recent decades. One of the most serious complications of T2DM is diabetic cardiomyopathy (DCM), an often underrecognized yet severe condition that is a leading cause of mortality among diabetic patients. In the early stages of DCM, patients typically show no symptoms and maintain normal systolic and diastolic left ventricle function, making early detection challenging. Currently available clinical markers are often not specific enough to detect the early stage of DCM. Conventional biomarkers of cardiac mechanical stress and injury, such as natriuretic peptides (NPs) and cardiac troponin I (cTnI), have shown limited predictive value for patients with T2DM. NPs have proven efficacy in detecting diastolic dysfunction in diabetic patients when used alongside 2D echocardiography, but their utility as biomarkers is limited to symptomatic individuals. While cTnI is a reliable indicator of general cardiac damage, it is not specific to cardiac injury caused by high glucose levels or T2DM. This underscores the need for research into biomarkers that can enable early diagnosis and management of DCM to reduce mortality rates. Promising novel biomarkers that showed good performance in detecting diastolic dysfunction or heart failure in diabetic patients include galectin-3, ST2, FGF-21, IGFBP-7, GDF-15, and TGF-β. This review summarizes the current understanding of DCM biomarkers, aiming to generate new ideas for the early recognition and treatment of DCM by exploring related pathophysiological mechanisms.

## 1. Introduction

Diabetes mellitus (DM) is an important risk factor for cardiovascular disease, representing a frequent cause of micro- and macrovascular complications, cardiac damage including ischemic coronary artery disease, diabetic cardiomyopathy (DCM), and autonomic neuropathy, with a cardiovascular mortality rate twice as high as in non-diabetic patients [1]. According to the latest projections from the International Diabetes Federation (IDF), the prevalence of DM in adults is currently around 10.2%. The global diabetes population is expected to increase to 578 million by 2035 and is anticipated to reach 700 million by 2045 [2]. DCM has previously been defined by the American College of Cardiology Foundation, the American College of Cardiology, the European Society of Cardiology, and the European Association for the Study of Diabetes as a structural and functional alteration without the presence of confounding factors, such as arterial hypertension, ischemic heart disease, and important valvopathy, in diabetic patients [3,4]. Accurately defining this disease is quite challenging, given that most patients with type 2 diabetes mellitus (T2DM) also have cardiovascular disease (CVD) [5,6]. The latest consensus statement of HFA/ESC from 2024 proposes the term “diabetic myocardial disorder” and a new definition for DCM. Therefore, this entity should be defined as systolic and/or diastolic myocardial dysfunction in the presence of T2DM. T2DM is seldom the only etiological factor for myocardial dysfunction, typically contributing in conjunction with obesity, arterial hypertension, ischemic heart disease, and chronic kidney disease, thereby leading to cumulative myocardial impairment. There is still a lack of evidence concerning the clinical phenotypes and the evolution of stages in this disease, despite thorough investigation, because there are no sufficient clinical data or the data are derived from experimental evidence [7]. A previous proposed classification of DCM [8] is detailed in Table 1.

In the early stages, it includes a subclinical period defined by structural and functional changes, including left ventricular hypertrophy, fibrosis, and alterations in cell signaling. These pathophysiological changes often evolve into heart failure [5,6].

The first two stages consist of phenotypes attributable only to diabetes, while the latter two include additional pathologies. The first stage consists in diastolic dysfunction, preserved ejection fraction, in the absence of other causes of cardiac disease. This mixed, hypertrophic, and restrictive phenotype is the earliest form and can be detected in 75% of asymptomatic patients. In stage 2, both diastolic and systolic dysfunction are present in the absence of other risk factors. Stages 3 and 4 present clinically manifest cardiac disease against the background of micro- and macrovascular damage [8].

Epidemiological data demonstrate a significant correlation between DM and heart failure (HF) [9]. Patients with T2DM have an elevated risk of developing HF, with a prevalence ranging between 19% and 23% [10,11,12], and the relationship is reciprocal. Echocardiographic studies indicate that 40–60% of asymptomatic diabetic patients exhibit diastolic dysfunction. Furthermore, diabetic patients with subclinical diastolic dysfunction face a higher 5-year mortality risk compared to those without diabetes and diastolic dysfunction (30.8% vs. 12.1%) [13,14]. The risk of heart failure is closely linked to long-term glucose management. Specifically, the risk of heart failure increases by 8% in individuals with T2DM for every 1% rise in hemoglobin A1c levels and by 30% for patients with type 1 diabetes [15].

Research examining the incidence and predictive significance of DCM among community-dwelling patients found that over a five-year period, individuals with DCM were at a significantly higher risk of developing HF compared to those with normal blood glucose levels, regardless of the severity of their condition [16]. The Framingham study further highlighted a 2.4-fold increase in HF incidence among diabetic men and a 5-fold increase among diabetic women. Additionally, HF is more commonly the first manifestation of cardiovascular disease (CVD) in individuals with DM compared to myocardial infarction [9,17].

DM is an independent predictor of cardiovascular morbidity and mortality in patients with chronic symptomatic HF, whether they have HF with preserved ejection fraction (HFpEF) or reduced ejection fraction (HFrEF), underscoring its consistent diagnostic significance across both categories [18]. The relationship between DM and HF is bidirectional: DM worsens outcomes for HF patients, and those with DM are at an increased risk of developing HF. Given the strong epidemiological link between HF and DM, HF should be recognized as a critical cardiovascular outcome for assessing the effectiveness of new glucose-lowering therapies [19].

Because DCM is an increasingly recognized entity, a good understanding of the pathophysiological mechanisms is required for early diagnosis and the development of therapeutic strategies to reduce the risk of developing heart failure in diabetic patients [20].

DCM represents a unique pathophysiological condition that is difficult to detect through routine clinical examination because early-stage DCM often lacks noticeable clinical symptoms or signs. As a result, DCM is generally diagnosed only after some form of cardiac dysfunction has already occurred, which can severely impact the management and treatment of DCM patients. The structural and functional cardiac impairments that accompany the presence of T2DM are frequent, but they are not specific and there is no “gold standard” for diagnosis. It was suggested that the criteria for diagnosis in diabetic myocardial disorder should comprise at least left ventricular diastolic dysfunction, and/or a decrease in left ventricular ejection fraction, a hypertrophic LV, and interstitial fibrosis [7]. Structural modifications such as LV hypertrophy or interstitial fibrosis may be detected through non-invasive methods such as echocardiography, magnetic resonance, or computed tomography, or by an invasive method such as endomyocardial biopsy. Functional alterations like diastolic and systolic dysfunction are discerned via echocardiography using tissue and pulsed wave Doppler imaging, 2D-speckle-tracking echocardiography for assessing global longitudinal strain, and stress echocardiography. The evaluation of metabolic alterations in the diabetic myocardium is achievable through proton magnetic resonance spectroscopy (1 H-MRS) to identify an increase in myocardial steatosis, as well as through single-photon emission computed tomography and positron emission tomography, which can reveal modifications in myocardial glucidic and fatty acid consumption. In clinical practice, echocardiography is used as it is a non-expensive and non-invasive tool [21].

Research indicates that cytokines, including various serum biomarkers, undergo significant changes during the early stages of DCM development, suggesting their potential use in early diagnosis. Therefore, it is essential to identify biomarkers with high specificity and sensitivity to establish an effective method for early DCM diagnosis.

In this review, we aim to present an up-to-date summary of the current knowledge regarding the mechanism of action and the role of conventional and novel cardiac biomarkers in early detection of DCM and its prognosis. Therefore, we performed research using Pub Med, Cochrane, and MEDLINE databases in order to analyze the most relevant studies describing biomarkers that fulfill our objective.

## 2. Pathophysiological Mechanisms

The pathophysiological mechanisms of diabetic cardiomyopathy are not fully elucidated, which is thus a concern of the scientific community. Many studies negate its existence, given that the cardiovascular morbidity and mortality of diabetic patients is mainly due to coronary heart disease related to the existence of risk factors such as dyslipidemia and hypertension [7,20,21]. There are a great many theories that probably influence the appearance of this disease [4]. DCM was confirmed as a new clinical entity in the early 1970s, after postmortem studies were carried out on four patients who had both type 2 diabetes and HF. DCM is associated with left ventricular hypertrophy along with either systolic or diastolic dysfunction, which can be in the form of HF with preserved ejection fraction (HFpEF) and fatty acid (FA) accumulation in cardiomyocytes [3,15].

The prevalence of DCM is rising in tandem with increasing rates of DM. This condition initially manifest with myocardial fibrosis, impaired remodeling, and accompanying diastolic dysfunction, eventually advancing to systolic dysfunction and finally overt heart failure. Several factors contribute to the development and progression of diabetic cardiomyopathy, including impaired cardiac insulin metabolic signaling, mitochondrial dysfunction, increased oxidative stress, reduced nitric oxide bioavailability, elevated levels of advanced glycation end-products, stiffness in cardiomyocytes and the extracellular matrix due to collagen, impaired mitochondrial and cardiomyocyte calcium handling, inflammation, activation of the renin–angiotensin–aldosterone system, cardiac autonomic neuropathy, endoplasmic reticulum stress, microvascular dysfunction, and various cardiac metabolic abnormalities [5].

**Figure 1:**
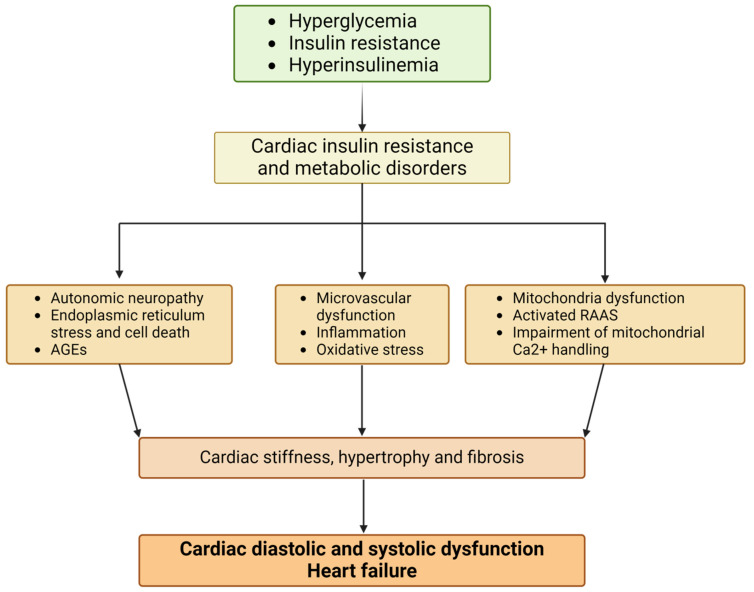
Pathophysiological mechanisms of diabetic cardiomyopathy (figure created with Biorender.com). Abbreviations: AGEs, advanced glycation end-products; RAAS, renin–angiotensin–aldosterone system; Ca, calcium.

The main pathophysiological mechanisms proposed to explain the cardiac changes include hyperglycemia, insulin resistance, hyperinsulinism, oxidative stress, myocardial lipotoxicity, autonomic neuropathy, mitochondrial dysfunction, activation of the renin–angiotensin system, and myocardial fibrosis [5,7,20], as illustrated in Figure 1.

Hyperglycemia is recognized as a critical factor in the development of DCM, as it activates various mechanisms that contribute to the disease’s progression. Hyperglycemia plays a significant role in both macrovascular and microvascular damage. Beyond its atherosclerotic effects, glucose contributes to a range of disease-related end-organ impacts, many of which involve vascular damage [20].

There is a considerable body of evidence pointing out that insulin manifests a varied influence on the onset of DCM as it modifies a range of cell mechanisms, which encompass glucose transportation, glycogen production, glycolytic actions, LV hypertrophy, synthesis of proteins, and lipid metabolic functions. Additionally, insulin is essential in modulating myocardial contractile function and provides defense against ischemic myocardial injury, autophagic processes, and cell survival, in a direct manner or through the action of insulin-like growth factor 1 (IGF-1) [22].

Oxidative stress and the accumulation of reactive oxygen species (ROS) play crucial roles in the development of diabetic complications and vascular disease, potentially serving as the primary trigger in the cascade of diabetic vascular pathology. Endothelial and vascular smooth muscle cells are particularly susceptible to oxidative damage, which affects lipids, proteins, and nucleic acids. One of the most important changes in diabetic patients is impaired endothelial function, primarily due to the loss of nitric oxide bioactivity [20,23].

Brownlee et al. demonstrated that hyperglycemia leads to an overproduction of superoxide by the mitochondrial electron transport chain [24]. Additionally, diabetes is associated with myocardial cell death, but it remains unclear whether this is due to a direct toxic effect of high glucose levels on myocytes or if it occurs through the activation of other pathways that produce cellular necrosis and apoptosis.

The energy needed for the functioning of a healthy heart comes in equal proportions from glucose and free fatty acid (FFA) metabolism. However, in hyperglycemic states, FFAs are used as the primary substrate for energy production. This shift is due to insulin resistance and the decreased transcription of glucose transporters in the myocardium, both of which lead to reduced glucose uptake by myocytes. To meet its energy demands, the myocyte turns to FFA metabolism, which unfortunately generates various toxic metabolites that accumulate within the myocytes, leading to lipotoxicity [20,25]. Lipotoxicity causes a disturbance in calcium regulation, which causes an accumulation of calcium ions in the cell, leading to ventricular stiffness seen in the early stages of DCM. Lipotoxicity is linked to the elevated production and release of reactive oxygen species (ROS), leading to oxidative stress and abnormal gene expression. This process ultimately results in cardiomyocyte death, myocardial fibrosis, and dysfunction [20].

T2DM patients have elevated concentrations of the angiotensin II receptor, and the activation of the renin–angiotensin system leads to oxidative stress that causes a disturbance in the balance between the production of reactive oxygen species and antioxidant defense, cellular apoptosis, and eventually fibrotic processes [20].

Glucose interacts with collagen deposition, forming Schiff bases, which are reorganized into glycated collagen and finally into advanced glycation end-products (AGEs), causing myocardial fibrosis and diastolic disfunction [26].

**Figure 2:**
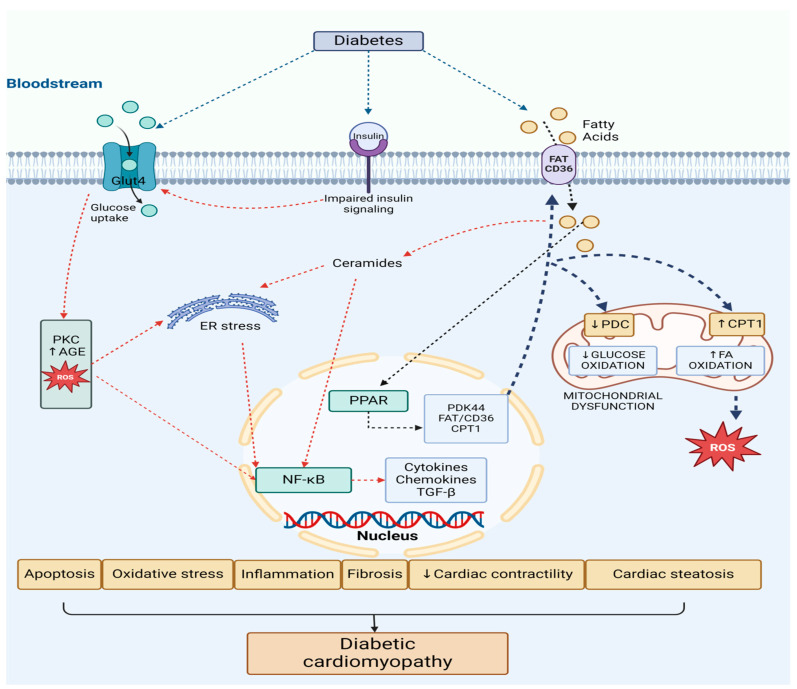
Pathophysiological mechanisms of diabetic cardiomyopathy—molecular basis (figure created with Biorender.com). Abbreviations: FA—fatty acids, FAT/CD36—fatty acid translocase, GLUT 4—glucose transporter 4, PPAR—peroxisome proliferator-activated receptor, CPT1—carnitine palmitoyl-transferase 1, PDK4—pyruvate dehydrogenase kinase 4, PDC—pyruvate dehydrogenase complex, ROS—reactive oxygen species, NF-κB—nuclear factor-kB, AGEs—advanced glycation end-products, PKC—protein kinase C, ER—endoplasmic reticulum.

An extensive molecular representation of pathophysiological mechanisms of DCM is illustrated in Figure 2.

There is an increase in free fatty acids (FAs) and glucose plasma levels triggered by diabetes. They are taken up by cardiac cells through both FA translocase (FAT/CD36) and glucose transporter 4 (GLUT4). The surplus of FA levels activates the peroxisome proliferator-activated receptor (PPAR) and its coactivator PGC-1α pathway. The consequence of this action is an enhanced expression of genes that promote FA uptake and oxidation, such as carnitine palmitoyl-transferase 1 (CPT1) and FAT/CD36. Additionally, the PPAR pathway induces the expression of pyruvate dehydrogenase kinase 4 (PDK4), which inhibits the pyruvate dehydrogenase complex (PDC), therefore reducing glucose oxidation.

This fact diminishes the assimilation and utilization of the glucose and shifts to FA oxidation in the mitochondria. This shift towards an increased FA oxidation causes a detrimental metabolic flexibility and energy production (ATP) by the heart because of the mitochondrial dysfunction, and leads to elevated production of reactive oxygen species (ROS) by the mitochondria [27].

Simultaneously, insulin resistance down-regulates insulin signaling, preventing GLUT4 translocation to the cell surface, thereby reducing glucose uptake and usage. This promotes a metabolic shift towards greater mitochondrial FA β-oxidation.

Even though the FA oxidation process is enhanced, myocardial lipid accumulation (cardiac steatosis) still occurs. Consequently, this leads to the synthesis of toxic lipid intermediates like ceramides and diacylglycerol (DAG) all leading to progression to heart failure. Proinflammatory transcription factors such as NF-κB and activator protein-1 (AP-1) are activated, contributing to endoplasmic reticulum (ER) stress and mitochondrial dysfunction [27].

Moreover, hyperglycemia promotes the development of reactive oxygen species (ROS) and advanced glycation end-products (AGEs) at the cardiac level. NF-κB is further activated, which leads to inflammation, cytokine and chemokine production, and interstitial fibrosis through matrix metalloproteinase (MMP) and transforming growth factor β (TGF-β). The accumulation of ROS also accelerates apoptosis and exacerbates ER stress, disrupting calcium handling and ultimately reducing cardiac contractility, resulting in cardiac dysfunction [27].

## 3. Biomarkers in Diabetic Cardiomyopathy

Several pathophysiological mechanisms in the development of diabetic myocardial disorder, such as cardiac fibrosis processes, inflammatory response, cardiomyocyte apoptosis, oxidative stress, and metabolic dysregulation are represented by various cardiac biomarkers [28]. The disclosure of novel biomarkers to integrate these processes is of great interest for early diagnosis, the risk stratification of diabetic cardiomyopathy, and also their utility as a future therapeutic target for the prevention of disease progression.

The most important biomarkers representing different pathophysiological pathways involved in DCM are detailed in Table 2.

### 3.1. Biomarkers of Cardiac Damage

#### 3.1.1. Natriuretic Peptides

The pathological hypertrophy is described by the augmentation of cardiomyocytes, amplification of protein production, and reactivation of the expression of fetal genes, including β-myosin heavy chain (β-MHC) and the natriuretic peptides type A and type B (ANP and BNP). These neurohormones are synthesized and released by cardiac cells in the course of heart failure in order to mitigate volume and pressure overload by means of their vasodilatory and natriuretic effects [29]. As a result, circulating levels of ANP and BNP, together with the biologically inactive precursor N-terminal fragment of BNP (NT-proBNP), are commonly used as biomarkers for heart failure and myocardial infarction. These biomarkers could also play a valuable role in the diagnosis of DCM [22,71].

Natriuretic peptides (NPs), such as natriuretic peptide type A (atrial natriuretic peptide-ANP) and natriuretic peptide type B (brain natriuretic peptide-BNP), have been suggested as potential biomarkers for DCM in previous studies [33,72]. Specifically, BNP could serve as an affordable and easily accessible marker for detecting preclinical ventricular diastolic dysfunction in patients with type 2 diabetes mellitus (T2DM), as stated by Romano et al. [29]. Elevated plasma BNP levels have shown strong predictive capacity for left ventricular dysfunction and heart failure in DCM [22,34].

Recent studies that enrolled cohorts of diabetic patients have stated that N-terminal prohormone BNP (NT-proBNP) is a significant biomarker that may assist in the early detection of heart failure (HF) in T2DM in studies conducted by Lapi et al. [30] and Patel et al. [31]. Moreover, both natriuretic peptides have proven efficacy in detecting diastolic dysfunction in diabetic patients when used alongside 2D echocardiography [32,73]. However, the usefulness of these biomarkers is restricted to symptomatic individuals, especially those with a mitral flow pattern that is pseudo-normal or restrictive [32]. In patients without symptoms or with a mitral flow pattern of altered relaxation, there was a lack of significant correlation between the levels of NPs and diastolic dysfunction [32]. Further studies have also confirmed that in asymptomatic patients there is no correlation between NPs and diastolic dysfunction, as well as a generally poor correlation with most echocardiography parameters [74,75]. In summary, while the usefulness of natriuretic peptides in detecting pre-clinical DCM is limited, BNP remains an independent predictor of poor outcomes in this cardiomyopathy [29,35,36]. In patients with type 1 diabetes mellitus (T1DM), ANP, rather than BNP, seems to be a more sensitive biomarker for early diastolic dysfunction [33].

It remains a topic of debate whether NPs have a specific role in the initial stages and progression of DCM. In obese, insulin-resistant, and T2DM individuals, there are studies that indicate low levels of NPs and impaired ANP and BNP signaling [76], while other studies report the opposite [77]. These discordant results can be attributed to the suggested two-phase connection between BNP and T2DM. It is stated that there is a lower risk of diabetes when BNP levels are normal, but the risk increases when BNP levels rise in pathological conditions such as myocardial infarction and HF [27].

#### 3.1.2. Cardiac Troponin I (cTnI)

Another well-established family of cardiac markers includes the troponins, a set of proteins that regulate the calcium-mediated interaction between actin and myosin. This multiprotein complex comprises troponin C, which binds calcium; troponin T (TnT), which binds to tropomyosin; and troponin I (TnI), which inhibits actin–myosin interaction [78]. Cardiac troponin I and T are widely used in routine clinical practice due to their high sensitivity and specificity for detecting myocardial injury [79]. Research in both humans and animals suggests that TnI and TnT are constitutively phosphorylated in diabetes through the action of protein kinase C (PKC), leading to impaired myofilament function and reduced Ca^2+^ responsiveness [80]. In patients with diabetes, TnT and TnI levels are elevated, especially in those with concomitant ischemic heart disease. However, no studies have explored the differences in troponin plasma levels between T2DM patients with DCM and those without [40,41].

In asymptomatic T2DM subjects, there is evidence that increased levels of cardiac troponin I (cTnI) serve as a marker of cardiac damage [37,38]. At present, cTnI is considered one of the circulating markers for identifying cardiac injury in T2DM individuals, alongside C-reactive protein (CRP) and electrocardiography (EKG) [39].

Despite the fact that cTnI is a solid indicator of general cardiac damage, it is not specific to cardiac injury triggered by diabetes and therefore cannot be regarded as a specific biomarker for DCM. Nonetheless, cTnI is still considered a useful diagnostic tool for screening asymptomatic diabetic patients for emerging heart failure (HF) risk [81].

#### 3.1.3. O-Linked N-Acetylglucosamine (O-GlcNAc)

Increased levels of O-linked N-acetylglucosamine (O-GlcNAc) have been shown to be correlated with the detrimental cardiac effects of diabetes, involving reduced contractility, impaired calcium handling, and an altered response to stress [81,82]. O-GlcNAcylation has also been linked to the progression of cardiovascular dysfunction in T2DM [83]. The harmful alteration of proteins by O-GlcNAc works as a significant modulator of the diabetic phenotype in human hearts. Therefore, reestablishing the functional equilibrium of O-GlcNAc in the heart might provide a novel therapeutic option for treating heart failure induced by T2DM [81,84].

### 3.2. Biomarkers of Inflammation and Fibrosis

Biomarkers that can play an important role in identifying the initial stages of DCM comprise serum inflammatory agents like interleukins, TNF-α, and C-reactive protein (CRP); increased fibrosis-related biomarkers such as transforming growth factor-beta (TGF-β) and insulin-like growth factor binding protein 7 (IGFBP-7); and diminished antioxidant agents such as adiponectin and leptin [42]. A multi-marker approach consisting of advanced glycation end-products (AGEs), TNF-α, IL-6, creatinine, and insulin has been recommended for the initial recognition of diastolic dysfunction in diabetic patients [85].

#### 3.2.1. Transforming Growth Factor-β (TGF-β)

Transforming growth factor-β (TGF-β) is a cytokine implicated in the process of fibrosis, which is widespread in multiple tissues, with a role in stimulating extracellular matrix deposition [86]. TGF-β is up-regulated in patients with diabetes as a result of increased ROS production [43] and it correlates with the degree of cardiac fibrosis [87]. Notably, while the majority of TGF-β-induced cardiac fibrosis occurs through the modulation of fibroblast phenotype and function [88], another contributing mechanism is TGF-β-mediated induction of EndoMT [89,90], a harmful process implicated in the pathophysiology of HFpEF [91].

#### 3.2.2. Insulin-Like Growth Factor Binding Protein-7 (IGFBP-7)

Insulin-like growth factor binding protein-7 (IGFBP-7), a component of IGFBP superfamily, stands out as a potential biomarker of DCM. It modulates the signaling pathway of insulin growth factor (IGF) by interacting with insulin and IGFs [92]. IGFBP-7 binds with low affinity for IGF [93]. However, owing to its solid binding affinity to insulin (unlike IGFBP 1–6), IGFBP7 can influence insulin’s physiological activity, possibly contributing to insulin resistance, facilitating the progression of diabetes, as demonstrated by several studies [46,47]. It has been shown that in T2DM patients, particularly in those with diastolic dysfunction, serum levels of IGFBP-7 and TGF-β are increased [42].

In diabetic patients, the level of IGFBP-7 is positively correlated with increased collagen accumulation, fibrosis processes, and myocardial hypertrophy [42]. Confirming this, systematic proteomic research places IGFBP-7 as a biomarker involved in cardiac hypertrophy and HF [94].

Ghandi et al. [44] aimed to investigate the relationship between IGBP-7 and echocardiographic parameters of diastolic dysfunction in patients with HFpEF. Elevated levels of IGFBP-7 had a modest correlation with diastolic dysfunction and demonstrated a similar performance and discrimination capacity to that of natriuretic peptide B [44]. In another study conducted by Ghandi et al. including patients with HFrEF, IGFBP7 was significantly correlated with the presence and severity of echocardiographic parameters of abnormal diastolic function [45]. The study reveals that raised concentrations of IGFBP-7 in serial measurements are associated with worsening of diastolic function, increasing left atrial volume index (LAVi) or right ventricle pressure (RVSP), placing it as a novel prognostic biomarker for HFrEF [45].

Fibrosis biomarkers IGFBP-7 and TGF-β, and inflammation biomarkers IL-6 and TNF-α, are notably elevated in patients with diastolic dysfunction. The most compelling evidence for the utility of IGFBP7 and TGF-β with regard to DCM was presented by Shaver et al. [42], who investigated multiple serum biomarkers in a population from West Virginia. The study assessed a comparison between the levels of circulating biomarkers in diabetic patients vs. control groups, and with a particular focus on diabetics with diastolic dysfunction versus those without diastolic dysfunction. IGFBP7 TGF-β, IL-6, and TNF-α plasma levels were significantly higher in diabetic patients with diastolic dysfunction, placing them as promising candidates for the early detection of DCM. [42].

#### 3.2.3. Growth Differentiation Factor-15 (GDF-15)

GDF-15 is a component of the TGF-β superfamily, which is generated as a reaction to oxidative stress and inflammatory processes by a variety of cell types consisting in macrophages, fat tissue cells, and cardiovascular cells [95]. It is highly expressed in the heart and plays a role in suppressing the production and release of cytokines that play a role in inflammation, specifically TNF-α and IL-6, along with controlling cell growth and differentiation [96]. Diabetic patients have elevated circulating levels of GDF15. Moreover, high serum levels exhibit a positive association with baseline glucose and triglycerides levels, obesity, insulin resistance, and C-reactive protein (CRP), a marker of inflammation [71]. Multiple studies have proven that GDF-15 synthesis is higher in a prediabetic state and in diabetic patients as opposed to individuals without this conditions, placing GDF-15 as a promising biomarker for uncovering DCM and its implications in diabetic patients [48,49]. Additionally, higher levels of GDF-15 are independently associated with cardiac remodeling and poor prognosis in heart failure and atrial fibrillation [51,52]. An increase in GDF-15 levels over the course of a year is independently linked to a higher risk of future CV mortality [53] and elevated GDF-15 levels are strongly correlated with a higher all-cause mortality rate in patients with coronary artery disease (CAD) and peripheral artery disease (PAD) [54].

Additionally, several studies have explored the role of GDF-15 in diastolic dysfunction [97,98]. Baessler et al. showed that in subjects with morbid obesity, GDF-15 levels seem to better correlate with left ventricle diastolic dysfunction than NT-proBNP levels and that the addition of GDF-15 to NT-proBNP and established risk factors improves reclassification for the diagnosis of diastolic disfunction with possible heart failure, and adds incremental value to NT-proBNP [97]. Another study reveals GDF-15 correlation with multiple parameters of echocardiographic diastolic function and with the effort capacity at the 6 min walk test. When a classification for HFpEF did not include NT-proBNP as a diagnostic criterion, GDF-15 showed superior diagnostic properties for detecting HFpEF compared to NT-proBNP. Moreover, combining GDF-15 with NT-proBNP significantly improved diagnostic accuracy [98].

In a study conducted by Dominguez-Rodriguez et al. [50], elevated GDF-15 levels demonstrated a good prediction for the development of DCM even in the absence of other risk factors, such as age, smoking, hypertension, and known cardiovascular disease. Importantly, a novel pharmacological agent aimed to target the GFRAL (high-affinity binding receptor for GDF-15/RET (receptor tyrosine kinase) pathway is currently under development for the therapeutic management of obesity and metabolic syndrome, which may have the ability to mitigate cardiovascular risk in patients with metabolic diseases by enhancing the underlying effects of GDF-15 [99].

However, its effectiveness in predicting disease progression, prognosis, or cardioprotection is limited by its lack of specificity for metabolic diseases and its association with an increased incidence of cancer in patients with T2DM [96].

#### 3.2.4. Cardiotrophin-1 (CT-1)

Cardiotrophin-1 (CT-1) is a constituent of the IL-6 cytokine family and is generated by cardiomyocytes and cardiac fibroblasts, and, as a consequence, is correlated with situations that imply mechanical stress as well as metabolic and hypoxic damage [100]. CT-1 activates the gp130 receptor, which enables the enhancement of cellular longevity by inactivation of proapoptotic Bcl-2-associated death promoter (BAD) protein. The BAD protein is inactivated through the phosphorylation of the PI3K/Akt pathway [100].

Should the stressful processes be sustained, CT-1 contributes to initiation of harmful processes such as scar tissue formation, myocardial remodeling, and hypertrophic response. All these impairments ultimately culminate in myocardial dysfunction and heart failure [100]. CT-1 facilitates the hypertrophic response of cardiomyocytes through the JAK/STAT3 (janus kinase/signal transducer and activator of transcription 3) and ERK5 (extracellular signal-regulated kinase 5) pathways, while it enhances cardiomyocyte survival by activating the p42/44 MAPK and PI3K/Akt pathways [101].

Camella-Pozuela et al. showed that plasma CT-1 levels are higher in patients with type 2 diabetes (T2DM) or impaired glucose tolerance compared to controls, with positive correlations with glycemia, blood pressure, and left ventricular hypertrophy, decreased ankle-brachial index, and microalbuminuria [62,102].

A significant body of evidence indicates that alterations in left ventricular (LV) geometry, the development of LV hypertrophy, and both systolic and diastolic heart failure (HF) are linked to reduced expression of CT-1 receptors in the heart and elevated levels of CT-1 circulating in the peripheral blood [103]. Moreover, the level of circulating CT-1 serves as a predictor for the risk of T2DM complications, T2DM-related organ damage, and cardiovascular (CV) events, independent of traditional CV risk factors [62].

In obese subjects, diminished plasma concentrations of CT-1 seem to correlate with a lower likelihood of developing both metabolic syndrome and T2DM [104]. Despite the important function of CT-1 in the pathophysiological mechanism underlying DCM, there exist two primary constraints that impede its utility as DCM diagnostic tool [105]. Firstly, CT-1 is produced across a multitude of other tissues, which encompass the liver, lungs, kidneys, and striated muscles [106]. In addition, shifts in the plasma amounts of CT-1 are connected to other forms of cardiomyopathies, such as ischemic cardiomyopathy, which reduces its specificity as a biomarker for DCM [107].

#### 3.2.5. Galectin-3 (Gal-3)

Galectin-3 (Gal-3) is a lectin that binds to β-galactoside, which is considered a pivotal connector among inflammation, fibrosis, and detrimental cardiac remodeling in heart failure [56,108]. Gal-3 is distributed in the heart, brain, lungs, adipose tissue, and blood vessels. In organs that target vascular complications, Gal-3 is not expressed, or is only poorly expressed under basal conditions. However, increased expression of Gal-3 could be defined in several tissues in metabolic diseases, such as diabetes and obesity, contributing to tissue remodeling [109].

Given the fundamental role of cardiac fibrosis in the pathogenesis of DCM, Gal-3 may facilitate the advancement of DCM through diverse mechanisms. In heart failure experimental rodent models, it was shown that there are activated macrophages and fibroblasts that locally produce Gal-3. It exerts its profibrotic action by promoting the proliferation of myofibroblasts, the aggregation of the extracellular matrix, macrophage infiltration, and LV hypertrophy through the stimulation of the TGF-b signaling pathway [108,110].

Plasma levels of Gal-3 have been proposed as a good biomarker for prediction of left ventricular systolic dysfunction and HF in diabetic patients [111]. In a study assessed by Lebedev et al. in a population of T2DM patients, the results showed a strong prediction of heart failure, indicating higher levels of Gal-3 in patients with HFpEF and HFmEF compared to DM patients without HF [56].

Another study performed by Ramirez et al. stated that Gal-3 is useful in the early detection of DCM, especially when used in combination with the echographic assessment of global longitudinal strain (GLS), providing good sensitivity (Se) and specificity (Sp) [55]

Additionally, higher levels of Gal-3 have been observed in T2DM patients compared to non-diabetic individuals [112], placing Gal-3 as a viable tool for detecting the initial stages of cardiomyopathy in T2DM patients.

Regarding prognosis, there are various studies that enhance the importance of this molecule. Boer et al. performed some of the first trials evaluating Gal-3 levels in patients with HFrEF and HFpEF, concluding that for a follow-up period of 18 months, Gal-3 has a strong and independent predictive value for all-cause mortality and hospitalization in patients with HFpEF compared to patients with HFrEF. The plasma level of galectin was evaluated at 3 to 6 months, and a similar increase resulted in a more pronounced increase in mortality and hospitalization risk in patients with HFpEF compared to those with HFrEF [113].

Qi-hui et al. enrolled 284 diabetic patients and also demonstrated that galectin-3 was higher in patients with T2DM compared to patients in the control group. Elevated Gal-3 concentrations correlated with a high risk of micro- and macrovascular complications, HF, nephropathy, and peripheral arterial disease in patients with T2DM [57]. Tan et al. analyzed a cohort of T2DM patients demonstrating that high levels of Gal-3 are associated with adverse CV outcomes such as non-fatal myocardial infarction, stroke, coronary revascularization, and mortality, independent of traditional risk factors [58]. Confirming evidence of a strong association between elevated levels of Gal-3 in prediabetic individuals and T2D, and an increased risk of major adverse cardiovascular events (MACEs) and all-cause mortality caused by endothelial dysfunction, plaque formation, vascular calcification, comes from another study, by Tan et al. [59].

In experimental studies, Gal-3 suppression prevented the synthesis of profibrotic and proinflammatory markers in multiple tissues, which suggests a possible therapeutic usefulness in DCM [114].

#### 3.2.6. Soluble ST2 (Suppression of Tumorigenicity)

The soluble form of suppression of tumorigenicity 2 (sST2) has the role of a decoy receptor for interleukin-33 (IL-33), which turns down the inflammatory response by means of the IL-33/ST2/sST2 pathway [115]. Consequently, the protecting influences of IL-33 in atherosclerosis and LV remodeling are diminished, since this pathway is an essential element of the autocrine/paracrine instruments that safeguard tissues from injury [116,117].

A study conducted by Fousteris et al. [60] demonstrated that patients with type 2 diabetes (T2DM) have higher plasma concentrations of sST2 compared to healthy individuals. Moreover, even higher sST2 levels were observed in patients with T2DM and grade I left ventricular diastolic dysfunction, an early sign of DCM and poor glycemic control. These findings suggest that sST2 may be associated with the early stages of DCM [60].

Nonetheless, there is still a matter of debate whether these elevated concentrations are a consequence, rather than a cause, of the inflammatory response noticed in the myocardium. Supporting the use of ST2 as a biomarker for DCM, plasma levels of this receptor have been independently linked to cardiovascular mortality in patients with heart failure and diabetes. Moreover, combining ST2 with troponin T significantly enhances its predictive accuracy [61]. Consequently, sST2 has been included in the 2017 ACC/AHA guidelines and it might be recommended for improvement of risk stratification in individuals with acute and chronic HF [118].

#### 3.2.7. FGF 21 (Fibroblast Growth Factor-21)

FGF21 is a polypeptide involved in regulating glucose homeostasis and lipid metabolism. Upon binding to FGF and beta-Klotho receptors, it activates the MAPK signaling pathway, leading to increased blood glucose levels and ketogenesis. In the heart, FGF21 and beta-Klotho expression levels are low. However, there is evidence suggesting that myocytes secrete FGF21 as an autocrine factor to protect the heart from adverse cardiac remodeling [63,119]. The expression of FGF21 in the heart is regulated by the protein deacetylase Sirt1 (sirtuin1). In environments high in sugar and fat, activation of the Sirt1 pathway triggers the secretion of FGF21 by the heart, which acts in an autocrine manner to protect cardiomyocytes from oxidative stress by promoting the expression of certain antioxidant genes (e.g., Ucp2, Ucp3, and Sod2) [120]. FGF21 plays a critical role in suppressing apoptosis in myocardial cells caused by oxidative damage, both in vitro and in vivo, by regulating apoptosis-related genes and the oxidoreductase system. This function is significant for research into FGF21’s antioxidant properties and its potential for preventing and treating cardiovascular and other diseases linked to oxidative stress injury [119].

The first study to establish a connection between elevated FGF21 levels and diastolic dysfunction in humans was conducted by Ruey-Hsing in 2016. This biomarker has been shown to be positively associated with T2DM, hypertension, the severity of heart failure, ischemic heart disease, and peripheral arterial disease. In terms of prognostic value, FGF21 is comparable to NT-proBNP in assessing the presence of diastolic dysfunction and predicting cardiovascular events within one year in patients with HFpEF [63,119].

Recent clinical and subclinical research has demonstrated that increased serum levels of FGF21 are closely linked with diabetic cardiomyopathy [119], placing it as a potential biomarker for this condition. In a small cohort of T2DM patients, comparing patients with HFpEF with controls without HF, Ianos et al. showed that FGF21 has the ability to diagnose HFpEF with a good specificity and sensibility [64]. Still, it remains a matter of debate whether increased serum FGF21 is a constituent promoting the pathogenesis of DCM or a central component involved in restoring the injury caused by this disorder. There is growing evidence that the administration of exogenous FGF21 generally provides a protective impact on cardiovascular disease. These findings suggest that FGF21 is not only a biomarker of cardiovascular risk, but is also efficient in conferring protection regarding cardiovascular disease and helps to mitigate the risk of DCM. Therefore, the beneficial involvement of FGF21 in the pathogenesis of diabetic cardiomyopathy is progressively acknowledged [119].

Recently, a growing body of evidence has demonstrated that FGF21 may be a promising therapeutic option for treating DCM, particularly in reducing oxidative stress [121], inflammation [121], apoptosis [122], and lipid accumulation [123] in the myocardium. For example, Wu et al. showed that FGF21 reduces inflammation in cardiomyocytes by upregulating the adenosine 5′-monophosphate (AMP)-activated protein kinase (AMPK)/paraoxonase 1 (PON1) signaling pathway [121]. Additionally, Zhang et al. found that FGF21 alleviates diabetes-related cardiac apoptosis by activating the extracellular signal-regulated kinase 1/2 (ERK1/2), mitogen-activated protein kinase 14 (p38 MAPK), and AMPK pathways in a mouse model of type 1 diabetes mellitus (T1DM) [122,124].

A clinical study conducted by Ong K et al. found that elevated baseline serum levels of FGF21 were associated with a higher risk of cardiac events in diabetic patients, suggesting that FGF21 could serve as a potential marker for early detection of cardiometabolic risk [66]. In a recent cross-sectional study, serum FGF21 levels were compared among prediabetic, diabetic, and healthy individuals. The study revealed that serum FGF21 levels were significantly higher in the prediabetic and diabetic groups compared to healthy controls. The cutoff value for diagnosing T2DM in this study had a sensitivity of 82.5% and a specificity of 60% [125]. Although the specificity of this biomarker is relatively low, it may still be useful for early screening of diabetic cardiomyopathy (DCM).

### 3.3. Biomarkers of Extracellular Matrix Remodeling: Matrix Metalloproteinases (MMPs) and Tissue Inhibitors of Metalloproteinase (TIMPs)

Matrix metalloproteases (MMPs) are calcium-dependent, zinc-containing endopeptidases that can degrade various extracellular matrix proteins and process several bioactive molecules. These enzymes are involved in cleaving cell surface receptors, releasing apoptotic ligands (such as Fas ligands), and inactivating chemokines and cytokines. MMPs are also believed to play significant roles in various cellular behaviors, including cell proliferation, migration, differentiation, angiogenesis, apoptosis, and host defense. Tissue inhibitor of metalloproteinase (TIMP) is a natural glycoprotein that inhibits MMPs [126,127].

In DCM, the accumulation of extracellular matrix proteins impairs cardiac contractility, leading to stiffness and the progression towards heart failure. As a result, monitoring extracellular matrix production and degradation may be used a valuable biomarker for detecting, diagnosing, and prevention of fibrosis process in DCM. In patients with DM, persistent hyperglycemia leads to the generation of oxidative stress (OS). Sustained hyperglycemia induces the synthesis of MMP-9, as evidenced by increased expression and activity of MMP-9 due to the oxidative stress generated in vascular endothelial cells [128,129].

For example, circulating levels of procollagen type 1 propeptide and MMP7 are associated with diastolic dysfunction in T2DM individuals. On the other hand, in animal studies with mice having type 1 or type 2 DM, who exhibit myocardial fibrosis and diastolic dysfunction, the MMP-2 levels are decreased [130]. Another MMP that is elevated in cardiac fibrosis and HF is MMP-9. In animal experiments including mice, the inhibition of MMPs using medication or genetic suppression has been shown to improve cardiac remodeling [91].

In hemodynamic models of heart failure with preserved ejection fraction (HFpEF), the activities of MMP2 and MMP9, along with TIMP1 protein levels, were increased, while metabolic models did not show changes in the mRNA expression of MMP2, -8, -9, -11, -14, and -15, or TIMP-1, -2, and -3 [127,131].

Ban et al. identified a relationship between serum MMP-7, diastolic disfunction, and the presence of diabetic microvascular complications. The study demonstrated that the level of MMP-7 was increased in patients with type 2 diabetes with diastolic dysfunction and in those with microalbuminuria [67].

### 3.4. Micro-Ribonucleic Acids (miRNAs) and Long Non-Coding RNAs (lncRNAs)

Micro-ribonucleic acids are also notable for their significant impact on regulating glucose uptake and cardiac metabolism in the diabetic heart [27].

Although miRNAs are small noncoding RNA molecules, they play a crucial role in modulating gene expression. Altered levels of miRNAs have been observed in the cardiomyocytes of experimental diabetes models. The expression of several miRNAs is influenced by matrix metalloproteinases (MMPs) [8,132].

miR-223 is associated with the regulation of glucose transporter 4 (GLUT4) expression in cardiomyocytes. Given that miRNAs function as stress response genes and are essential for maintaining the efficacy of physiological responses, such as restoring GLUT4 expression and normal glucose uptake, in the presence of pathophysiological conditions like insulin resistance, miR-223 is particularly noteworthy. It has the ability to upregulate target genes such as GLUT4 in adult cardiomyocytes, making it a potentially valuable therapeutic target [8,132].

Several microRNAs (miRNAs) and long non-coding RNAs (lncRNAs) have been highlighted as prospective biomarkers for DCM, as increasing evidence arises [133]. Specifically, miRNAs that are linked to pathological processes such as myocardial inflammation (like miRNA-21) oxidoreductive signaling (including miRNA-221, miRNA-146a, miRNA-34a, miRNA-210, miRNA-19b, miRNA-125b, miRNA-27a, and miRNA-155), cardiac hypertrophy (miRNA-221), and apoptosis (miRNA-34a, miRNA-125b, miRNA-146a, miRNA-155, miRNA-210, and miRNA-221) are of significant interest [133].

LncRNAs may also play a role in the development of cardiac hypertrophy and heart failure in DCM by regulating redox and inflammatory signaling. Specifically, lncRNAs such as H19 [134], NON-RATT007560.2 [135], Kcnq1ot1 [136], HOTAIR [137], and ANRIL [138] have been associated with cardiac remodeling in DCM by influencing cardiomyocyte apoptosis and oxidative stress.

### 3.5. Extracellular Vesicles (EVs)

Extracellular vesicles (EVs) are small particles released by cells into the extracellular environment carrying nucleic acids such as miRNAs, DNA, proteins, and lipids, which they transport to specific cells in order to regulate a variety of intracellular processes. EVs can derive from myocardial cells, endothelial cells, fibroblasts, and oncosomes. The classical nomenclature based on biogenesis includes exosomes, which are formed through the exocytotic process of the multivesicular body (MVB); microvesicles (ectosomes), which bud directly from the plasma membrane of the cell; and apoptotic extracellular vesicles, which are produced as a consequence of cellular disintegration during the process of apoptosis. The EVs are involved in the pathogenesis of DCM, which is related to cardiomyocyte death and hypertrophy, endothelial damage, inflammation and fibrosis, calcium dyshomeostasis, and senescence. The Hippo pathway is among the most pivotal signaling pathways implicated in the regulation of apoptosis and autophagy, and mammalian sterile 20-like kinase 1 (Mst1) is integral to this regulatory mechanism. In vivo experiments have demonstrated that an overexpression of Mst1 correlates with a decline in cardiac functional capacity alongside an enhanced insulin resistance. Increased concentrations of Mst1 protein have been identified within exosomes derived from cardiac endothelial cells. The uptake of these exosomes by cardiomyocytes may lead to diminished autophagic activity and an increase in apoptotic processes, particularly under conditions of elevated glucose. Mst1 also inhibits GLUT4 translocation causing insulin resistance and elevated reactive oxygen species, which induce apoptosis [139].

The process of cardiomyocyte apoptosis results in a weakening of the cardiac structure. Therefore, as a response, the remaining cardiomyocytes initiate compensatory hypertrophy. MicroRNAs found in exosomes have a substantial impact on a wide range of cellular activities, promote intercellular communication, and regulate cell survival. Cardiac fibroblasts release exosomes that carry pro-hypertrophic substances. These exosomes have been shown to upregulate the renin–angiotensin system pathway, contributing to cardiac enlargement. Suppressing the production of these exosomes can help mitigate angiotensin II-induced cardiac hypertrophy. Among the microRNAs associated with extracellular vesicles, miR-21-3p is of particular interest. Its levels are increased in response to hyperglycemia, and in animal experiments its pharmacological inhibition has been found to decrease cardiomyocyte enlargement [139].

In DCM the endothelial cells homeostasis is impaired because of the modulation of endothelial function and angiogenesis by miR-126, miR-320, and miR-503 contained in the EVs. The release of miRNA-503 from EVs reduces pericyte proliferation and migration, and decreases angiogenesis and vascular permeability. High levels of miR-320 restrain angiogenesis. Conversely, in hyperglycemia conditions the concentration of proangiogenic miR-126 is decreased and causes oxidative stress and apoptosis in endothelial cells [139].

The exosomes that derive from macrophages containing miR-155 increase the fibroblasts secretion of collagen and proinflammatory cytokines, and inhibit the anti-inflammatory genes. Additionally, they create calcium dyshomeostasis, resulting in diastolic and contractile dysfunction. Currently, clinical trials are investigating the diagnostic performance of EVs. Additionally, the preclinical data show beneficial results of EVs, such as anti-apoptotic effects, improved insulin sensitivity, anti-inflammatory and antifibrotic effects, and regulation of intracellular calcium concentration [139].

The knowledge regarding the most representative studies that included conventional and novel circulating biomarkers, emphasizing the future clinical utility in diagnosis and prognosis of diabetic cardiomyopathy is summarized in Table 3.

## 4. Conclusions

Diagnosing DCM in the initial stages is crucial in order to prevent the progression of irreversible structural modifications, especially fibrotic processes, which ultimately result in detrimental contractility and overt heart failure. While current diagnostic approaches, including echocardiography, levels of NT-proBNP, cardiac magnetic resonance, and nuclear imaging can detect DCM, these methods have limitations. Moreover, they tend to detect DCM at a later stage of the disease. The conventional biomarkers that represent myocardial damage and the ventricular hemodynamic overload does not seem to be specific for DCM.

There is growing interest in novel biomarkers that can identify early signs of cardiac functional changes. Galectin-3 (Gal-3) is a particularly promising biomarker, especially when used alongside others such as FGF-21, IGFBP, MMPs, TIMPs, miRNAs, lncRNAs, and TGF-β levels. These biomarkers can provide insight into critical aspects of the metabolic and functional status of the heart. Gal-3 is associated with insulin resistance, fibrogenesis, and extracellular remodeling, and is valuable in assessing profibrotic activity. FGF-21 plays a role in metabolic states like insulin resistance, lipid accumulation, and gluconeogenesis. The high synthesis of IGFBP-7 overexpression is associated with insulin resistance; TGF-β is responsible for the initiation of fibrotic processes; MMPs and TIMPs are associated with cardiac remodeling; and miRNAs control diverse pathways in the pathogenesis of DCM. In conclusion, natriuretic peptides (NT-proBNP, BNP) have high specificity in detecting diastolic dysfunction in diabetic patients, but there are also discordant results. There are some promising novel biomarkers, such as galectin-3, ST2, FGF-21, IGFBP-7, GDF-15, and TGF-β, that provide good specificity in diagnosing diastolic dysfunction or overt heart failure in diabetic patients. However, due to the paucity of studies, and the small number of studied patients, there are no current recommendations for their utility in clinical practice.

The alteration of these biomarkers at different stages of DCM indicates their potential utility in assessing disease progression, enabling clinicians to implement early interventions that could reduce mortality rates. Additional studies including larger cohorts of patients are needed to elucidate the optimal diagnostic and treatment strategies to mitigate the risks associated with DM.

## Figures and Tables

**Table 1:** DCM classification (adapted from Gilca et al.) [8].

Classification	Stage 1	Stage 2	Stage 3	Stage 4
Characteristics	Diastolic dysfunction, hypertrophy	Systolic dysfunction and dilatation	Systolic dysfunction, dilatation, HTA-associated	Including all confounding factors, also CAD
NYHA classification	Asymptomatic	NYHA II	NYHA II–III	NYHA II–IV
Metabolic status	Impaired glucose tolerance, metabolic syndrome	Chronic hyperglycaemia	Insulin resistance Presence of microangiopathic complications	Presence of micro- and macroangiopathic complications
Echocardiographic findings ± coronarography	Increased LV mass, diastolic dysfunction, decreased tissue velocities, normal EF	Increased LV mass and wall thickness, diastolic and systolic dysfunction, mild cavity dilatation	Diastolic dysfunction and mild systolic dysfunction, cavity dilatation	Moderate-severe systolic dysfunction, cavity dilatation, associated coronary artery disease
Related comorbidities			Microangiopathic complications; HTA	Macroangiopathic complications, including CAD

Abbreviations: NYHA—New York Heart Association, LV = left ventricle, HTA = hypertension, EF = ejection fraction, CAD = coronary artery disease.

**Table 2:** Biomarkers representing different pathophysiological pathways involved in DCM.

Biomarkers	Mechanism of Action	Clinical Significance in DCM	Supporting Evidence
**Biomarkers of cardiac damage**			
**NT pro BNP** **BNP** **ANP**	Are produced and secreted by cardiac cells during HF to combat volume and pressure overload through their vasodilatory and natriuretic effects	Show strong predictive capacity for left ventricular dysfunction and HF in DCM Positively correlated with insulin resistance in prediabetic individuals. Their utility is limited to symptomatic patients BNP—independent predictor of poor outcomes in DCM Predictors of new onset CAD and MACE Micro and macrovascular complications	Romano et al. [29] Lorenzo et al. [22] Lapi et al. [30] Patel et al. [31] Dalhstrom et al. [32] Bayerle-Eder et al. [33] Watson et al et al. [34] Brunner et al. [35] Rahimi et al. [36]
	
**hs-Tn I**	Inhibits actin–myosin interaction	Predictor of DCM Predictor of CV death and HF Independent predictor if MACE	Swoboda et al. [37] Babusha et al. [38] Marx et al. [39] Eggers et al. [40] Segre et al. [41]
**Biomarkers of inflammation and fibrosis**			
**TGF-b**	Modulates the fibroblast phenotype and function and mediates induction of EndoMT	Correlates with the extent of fibrosis Role in diagnosing early stage of DCM	Shaver et al. [42] Iglesias-De La Cruz et al. [43]
**IGFBP-7**	Modulator of insulin receptor activity and signaling, which displays a positive correlation with increased collagen deposition, fibrosis, and cardiac hypertrophy in diabetes	Correlates to cardiomyocyte hypertrophy and fibrosis Role in diagnosing early stage of DCM Marker of diastolic dysfunction and prognosis Potential role in causing insulin resistance and development of diabetes	Shaver et al. [42] Ghandi et al. [44] Ghandi et al. [45] Kutsukake et al. [46] LiuY et al. [47]
**GDF-15**	Member of the transforming growth factor β cytokine superfamily Regulates the inflammatory response, apoptosis, cell repair and cell growth, nergy homeostasis	Prediction of new-onset T2DM Prediction of DCM Prediction in HF and HF related outcomes Prediction of CV risk and mortality	Kempf et al. [48] BaoX et al. [49] Dominguez-Rodriguez [50] Kempf et al. [51] Wallentin et al. [52] Anand et al. [53] Hsu et al. [54]
**Galectin 3**	Enhances myofibroblast proliferation, the accumulation of extracellular matrix, and macrophage infiltration by stimulating the TGF-b (transforming growth factor b) signaling pathway. Interferes with insulin signaling by binding to the insulin receptor (IR)	Prediction of DCM Prediction of MACE and all cause mortality	Flores-Ramirez [55] Lebedev et al. [56] Qi-hui et al. [57] Tan et al. [58] Sun et al. [59]
**ST2**	A decoy receptor of IL-33. Elevated levels inhibit the protective effects of IL-33 in atherosclerosis and cardiac remodeling, by restricting the activation of the cascade initiated by the IL-33/ST2L interaction	Predictor of HF and HF related otcomes Predictor of CV disease and CV mortality	Fousteris et al. [60] Alonso et al. [61]
**Cardiotrophin-1 (CT-1)**	Promotes cardiac fibrosis and remodelling, and inhibits apoptosis, by activating the JAK/STAT3 and ERK5 pathways	Prediction of DCM	Camella-Pozuela et al. [62]
**FGF-21**	Role in regulating glucose homeostasis and lipid metabolism: insulin resistance, lipid accumulation, gluconeogenesis	Predictor of HF Predictor of CV events	Chou et al. [63] Ianos et al. [64] Lenart-Lipinska et al. [65] Ong et al. [66]
**Biomarkers of extracelular matrix remodeling**			
**MMP**	Enzymes involved in cleaving cell surface receptors, releasing apoptotic ligands, such as Fas ligands, and inactivating chemokines and cytokines	Prediction of DCM and microvascular complications	Ban et al. [67]
**miRNA and LncRNA**			
miRNA **LncRNA (LIPCAR, MIAT, SENCR)**	Modulation of gene expression Epigenetic regulation of multiple genes that are involved in diabetes and cardiac dysfunction	Associated with cardiac inflammation miRNA-21 redox signaling- imiRNA-221, miRNA-146a, miRNA-34a, miRNA-210, miRNA-19b, miRNA-125b,miRNA-27a, and miRNA-155) cardiac hypertrophy-miRNA-221 aapoptosis-miRNA-34a, miRNA-125b miRNA-146a, miRNA-155, miRNA-210 and miRNA-221 lncRNA LIPCAR is associated with Diastolic dysfunction lncRNA-MIAT associated with pathological angiogenesis	de Gonzalo-Calvo et al. [68] Yan et al. [69] Liu et al. [70]

Abbreviations: cTnI, cardiac troponin I; NT-proBNP, N-terminus of prohormone brain-derived peptide; O-GlcNAc = O-linked Nacetylglucosamine TGF-b—transforming growth factor b; IGFBP-7, insulin-like growth factor binding protein-7; MMP, matrixmetalloproteinase; JAK/STAT3—janus kinase/signal transducer and activator of transcription 3, sST2: Soluble form of suppression of tumorigenicity 2; GDF-15: Growth differentiation factor-15; TGF-β: Transforming growth factor-β; EndoMT—Endothelial–mesenchymal transition, CV-cardiovascular, MACE-major acute cardiovascular events, miRNA—microRNA; lncRNAs—long noncoding RNAs; LIPCAR—long intergenic non-coding RNA predicting cardiac remodeling, MIAT—Myocardial infarction-associated transcript, SENCR-smooth muscle and endothelial cell-enriched migration/differentiation-associated long noncoding RNA.

**Table 3:** Applicability of circulating cardiac biomarkers in T2DM patients.

Biomarkers	Subjects Included	Clinical Signification	References
NT-proBNP	7558 T2DM patients 6293 patients with DM: 4889 without ASCVD and 1404 with ASCVD 415 patients, 88 with DM, divided into 92 patients with diastolic dysfunction, 214 with LV normal function, 109 with LV systolic dysfunction 62 patients with T2DM grouped into 29 patients with HFpEF 13 patients with HfmEF 20 patients with DM, without HF 13 healthy controls	Association with ischemic cardiomyopathy, stroke, atrial fibrillation, hypertension	Lapi et al. [30]
		The use of a risk score (WATCH-DM) for developing HF, followed by NT-proBNP evaluation in those with a low risk, identified 84% of incident HF events over a 5-year follow-up BNP or NT-proBNP detect symptomatic patients with diastolic dysfunction with a restrictive filling pattern or pseudo-normalized mitral flow pattern. Asymptomatic patients may have normal levels of NP Higher values of NT-proBNP were noted in patients with HfmEF (*p* < 0.05)	Patel et al. [31] Dahlstrom et al. [32] Lebedev et al. [72]
BNP	127 T2DM patients without overt HF 76 T2DM asymptomatic patients: 39 with LVDD 23 with LV hypertrophy 1368 patients	Diastolic dysfunction was detected in 42% of cases. In an uncontrolled diabetic cohort, BNP was a strong predictor for LVDD at a cut-off value > 25 pg/mL, AUC = 0.8 with a Sp = 78%, Se = 77% No significant difference in BNP levels among patients with LVDD an patients with normal LV function AUROC = 0.53 In T2DM patients, BNP diagnosed Stage B pre-HF at a cut-off value ≥ 20 pg/mL, AUC = 0.75, with a Sp = 60%, Se= 80%	Romano et al. [29] Valle et al. [75] Watson et al. [34]
ANP	7 T1DM with diastolic dysfunction and 10 controls	Diastolic disfunction is associated with elevated ANP, but no with BNP	Bayerle-Eder et al. [33]
cTnI	130 asymptomatic T2DM patients: 50 patients with persistent microalbuminuria 50 patients without albuminuria 30 controls	cTnI levels were higher in DM with signs of diastolic dysfunction and microalbuminuria (*p* = 0.05) Cardiac extracellular volume fraction, a marker of diffuse fibrosis, assessed by MRI, was higher in DM patients (*p* = 0.0002) and in those with microalbuminuria (*p* = 0.004)	Swoboda et al. [37]
IGFBP-7 TGF-β IL-6 TNF-α	160 patients with HFpEF: 65 with T2DM 124 patients with HfrEF: 42 patients with DM 100 patients divided into 4 groups: DM with normal diastolic function DD without DM DM and DD control	A modest correlation between IGFBP-7 levels and echocardiographic parameters of DD, with a AROC = 0.68 for E/A > 1.5 and AUROC= 0.71 for E/e’ > 15 Similar performance to NT-proBNP (AUROC = 0.74 and 0.67) Elevated IGFBP-7 levels were associated with diastolic dysfunction Higher levels are predictive of an increased risk of CV events Raised concentrations in serial measurements are associated with worsening of diastolic function, increasing LAVi or RVSP Serum TGF-β, IGFBP-7, IL-6, and TNF-α were elevated in DM, DD and in patients with DM and DD, compared to the control group. Significantly higher in patients with DM + DD, when compared to DM or DD patients. *p* < 0.05	Ghandi et al. (2016-HFpEF) [44] Ghandi et al. (2014-HFrEF) [45] Shaver et al. [42]
GDF-15	213 T2DM patients: 45 patients with DCM, 168 without DCM 228 patients, 75 with T2DM: 142 patients with HFpEF 86 patients with HFrEF 18 controls	Patients with DCM had a higher level of GDF-15 compared to those without DCM. At a cut-off value of 3.81 pg/mL, the AUROC = 0.83 with a Se = 82.2%, Sp = 70.2%, *p* < 0.0001 Patients with HFpEF had significant higher levels of GDF15 than controls. Patients with HFpEF had similar values as patients with HFrEF. Correlation with echocardiographic parameters of diastolic dysfunction Role in diagnosis of HFpEF, AUROC = 0.89; Se = 81.7%, Sp = 85%. Using a combination between GDF15 and NT-proBNP improves diagnostic accuracy	Dominguez-Rodriguez [50] Stahrenberget al. [98]
Gal-3	121 T2DM asymptomatic patients: 76 patients with HFpEF and 14 with HFmEF 33 controls 1495 T2DM patients 62 patients with T2DM grouped into 29 patients with HFpEF 13 patients with HFmEF 20 patients with DM, without HF 13 healthy controls	Gal-3 was elevated in all DM patients vs. controls and higher in patients with HFmEF vs. HFpEF DM patients had lower GLS compared to controls At a cut-off value of Gal-3 > 2.71 ng/mL and GLS < 18%, a Se = 85% and a Sp = 81% was noted for LVD. Gal-3 is useful in the early detection of DCM High levels of Gal-3 were associated with adverse CV outcomes (non-fatal myocardial infarction, stroke, coronary revascularization) and mortality, independent of traditional risk factors, with a HR of 2.5 and 3.92 (*p* < 0.001) Higher values of Gal-3 were in HFpEF and HFmEF compared with DM patients without HF (*p* = 0.01, *p* = 0.03, respectively)	Flores-Ramirez et al. [55] Tan et al. [58] Lebedev et al. [56]
ST2	158 patients divided into 4 groups: 50 patients with DM and DD 48 patients with DM, without DD 18 patients without DM, with DD 42 controls 1069 patients: 385 patients with T2DM	Serum ST 2 was elevated in patients with DM compared to controls. Higher levels in patients with DM and DD compared to patients with DM without DD (*p* = 0.001) Role in diagnosing early stage of DCM ST 2 associated with CV and all cause mortality, with a HR of 1.27 and 1.23 (*p* < 0.001) in T2DM patients, during a follow-up of 5 years	Fousteris et al. [60] Alonso et al. [61]
FGF-21	87 T2DM patients 9795 patients with T2DM 238 patients: 95 with DD, 46 with DM 143 controls, 34 with DM 69 T2DM patients: 40 with HFpEF 29 without HF	Elevated FGF-21 levels > 240.7 pg/mL correlated with higher incidence of CV nonfatal events (myocardial infarction, heart failure, stroke, coronary revascularization) during a 2-year follow-up, with a HR of 4.7 Higher serum levels were associated with increased risk of CV outcomes, during a 5-year follow-up Correlation with echocardiographic parameters of diastolic dysfunction. AUROC = 0.665 in predicting DD FGF-21 was non-inferior to NT-proBNP to predict the presence of DD and CV events in 1-year follow-up FGF21 levels were significantly higher in HFpEF group with AUROC = 0.81,with a Sp = 79.3% and a Se = 85% *p* < 0.001, in predicting HFpEF, at a cut-off value of 217 pg/mL	Lenart-Lipinska et al. [65] Ong et al. [66] Chou et al. [63] Ianos et al. [64]
CT-1	93 patients with T2DM 209 hypertensive patients 82 controls	Diabetic or hypertensive patients have higher plasma levels of CT-1 compared to controls Correlation with LVH Correlation with CV events in 10-year follow-up	Gamella-Pozuelo et al. [62]
MMP-7	60 patients with T2DM 40 controls	Correlation between serum MMP-7, diastolic disfunction and the presence of diabetic microvascular complications. The level of MMP-7 was increased in patients with T2DM with DD and in those with microalbuminuria (*p* < 0.05)	Ban et al. [67]
LncRNA	48 patients with T2DM 12 controls	LIPCAR had positive association with grade I diastolic dysfunction (*p* < 0.050)	De Gonzalo-Calvo et al. [68]

Abbreviations: T2DM-type 2 diabetes mellitus, WATCH-DM risk score: Weight, Age, Hypertension, Creatinine, HDL-C, Diabetes Control, QRS Duration, Myocardial infarction and coronary artery bypass grafting (CABG), ASCVD—atherosclerotic cardiovascular disease, HF-heart failure, HFpEF—heart failure with preserved ejection fraction, HFmEF—heart failure with midrange ejection fraction, NP—natriuretic peptides, LV—left ventricle, DM = diabetes mellitus, DD = diastolic dysfunction, LAVi—left atrial volume index, RVSP—right ventricular systolic pressure, LVD—left ventricle dysfunction, GLS—global longitudinal strain, CV—cardiovascular, HR—hazard ratio, AUROC—area under ROC curves, LVH—left ventricular hypertrophy, MRI—magnetic resonance, LIPCAR—long intergenic non-coding RNA predicting cardiac remodeling.
